# Picture perfect? Gazing into girls’ health, physical activity, and nutrition through photovoice

**DOI:** 10.1080/17482631.2021.1874771

**Published:** 2021-01-25

**Authors:** Rebecca A. Spencer, Matthew Numer, Laurene Rehman, Sara F.L. Kirk

**Affiliations:** a School of Health & Human Performance, Dalhousie University, Halifax, NS, Canada; b Healthy Populations Institute, Dalhousie University, Halifax, NS, Canada

**Keywords:** Gender, photovoice, physical activity, nutrition, young adult, youth

## Abstract

**Background:** Women face contradictions regarding their health: Pressure to be feminine, but also athletic; Criticism for being too sporty or muscular, but equally so for being perceived as lazy or overweight. These complexities are perpetuated through media and discourse.

**Purpose:** Using a feminist post-structural approach and photovoice, this study explored health, physical activity, and nutrition in adolescent girls and young women.

**Methods:** Photovoice enables reflection, promotes dialogue, and sparks change. The process involved conducting a workshop, collecting photos, and participatory analysis sessions, which engaged the participants (n = 7, ages 13–26) in photo selection, contextualization, and codifying.

**Results:** This resulted in three themes: First, (Breaking) Stereotypes, in which participants identified gender norms, conflicts, and contradictions; Second, Emotional Safety, or the contexts in which girls and young women feel confident and comfortable; Finally, Being Outside in Nature emerged as significant. Each theme is supported by quotations and photographs. This work suggests being outside in nature provides important context for girls and young women to feel emotionally safe, such that they may engage in the complex navigation of competing discourses surrounding health.

## Introduction

Adolescent girls and young women face double-standards and contradictions regarding their bodies; they are pressured to be perceived as thin, feminine, and pretty, but are criticized if perceived as too athletic or muscular, and more so if perceived as overweight (Azzarito et al., 2006; Cockburn & Clarke, 2002; Evans, 2006; Flintoff & Scraton, 2001). These ideas are perpetuated through discourses of obesity and healthism (reinforcing health as individual responsibility); media promoting unrealistic body ideals and targeting by food industry; and relate to the health of girls and young women, and more specifically, their physical activity and nutrition (Brown & Witherspoon, 2002; Escobar-Chaves & Anderson, 2008; Sailors et al., 2012; Strasburger et al., 2010). As early as Greek times, women have been portrayed as weak and frail, with early Christianity’s portrayal of women as the temptress leading to the ongoing regular objectification of women, and the focus on their bodies that persists today (Butler, 1993; Duncan, 1994; Weitz, 2002). Women’s bodies are regulated by power relations and discourse, with some bodies being valued, and others being excluded (Bordo, 2003; Butler, 1993; Duncan, 1994).

These discourses are present against a backdrop of the United Nations’ Sustainable Development Goals, which have highlighted the importance of addressing gender issues (Health in 2015, 2015). Further, the World Health Organization and others have highlighted physical inactivity as a global concern, particularly for youth and women; in Canada, only 2% of girls aged 12–17 meet movement guidelines (Guthold et al., 2018; ParticipACTION, 2018; Roberts et al., 2017; World Health Organization, 2014). Canadian children are not sufficiently well-nourished, particularly regarding fruit and vegetable intake (Bushnik, 2016; Fung et al., 2013; Riediger et al., 2007). Nutrition is complex and tied to body image, with high rates of body dissatisfaction and unhealthy weight control among adolescent girls and young women (Boyce et al., 2008; Bushnik, 2016; Krebs et al., 2017; National Eating Disorder Information Centre, 2015; Patte et al., 2016). Collectively, the Global Strategy for Women’s, Children’s and Adolescents’ Health background document identifies physical activity as an emerging priority, nutrition as an area for necessary intervention, and the need for policy and environmental action to support the well-being of adolescent girls and young women (Temmerman et al., 2015).

Evidence indicates that complex problems require complex solutions (Kreuter et al., 2004; Signal et al., 2013; Ward et al., 2011). Literature consistently indicates that policies and interventions need to go beyond targeting individual behaviours to be multicomponent, multifaceted, and multilevel (Pate et al., 2011; Williams et al., 2013). To date, however, there has been limited national or international action. In Canada, individual provinces and school boards have adopted food and nutrition policies, though evidence for their efficacy is mixed, and there is currently a call for a national school food program (Fung et al., 2013; Hernandez et al., 2018; Kirk & Ruetz, 2018; Mâsse et al., 2014). Similarly, Canada has a recent “Common Vision” document, titled Let’s Get Moving intended to guide physical activity strategies nationally, and a recent national budget identified women, girls, and sport as a priority (Canadian Minister of Finance, 2018; Federal, provincial and territorial governments, 2018).

This work departs from a scoping review exploring what is known about gender norms, physical activity, and nutrition in adolescent girls. That review, published elsewhere, noted that girls and young women have complex relationships with physical activity; they are concerned about appearance and perceptions; body-focused discourse is significant; and highlighted the importance of social influences, institutions, and environments (Spencer et al., 2015). It also highlighted several gaps in the literature, including a lack of evidence exploring the experiences and perspectives of older adolescent girls and young women and a lack of inclusion of comprehensive approaches (Spencer et al., 2015).

A fundamental shift in the framing of popular discourses that problematize the bodies of girls and young women is required. This study addressed these complex concerns by using a participatory and feminist post-structural approach. The purpose of this study was to comprehensively explore the relationship between gender and adolescent girls’ and young women’s physical activity and nutrition. More specific objectives of this work included exploring how adolescent girls’ and young women’s perceptions of health, physical activity and nutrition, take up and contend with social, political, and cultural contexts; exploring how gender intersects with those perceptions; and exploring how the bodies of girls and young women are produced through the social, political, and cultural influences on health.

## Methods

### Theory: a feminist post-structural approach

This work employed a feminist post-structural approach. Feminist theory is focused on women, gender, political issues of oppression, and combating the patriarchy (Doucet & Mauthner, 2006; Hesse-Biber, 2012; Landman, 2006; Weedon, 1987). Post-structural theory explores the relations between power, language, and experience (Foucault, 1995). It can be used to expose and explore the production of and regulation of social narratives and norms (Barret, 2005; Numer & Gahagan, 2009; Weedon, 1987). Feminist post-structural approaches can therefore move beyond traditional research methods to explore discursive practices and political discourse as embodied experiences and explore how discourse maintains or challenges power relations (Azzarito et al., 2006; Barret, 2005; Weedon, 1987). Feminist post-structural approaches assume identity, or subjectivity, to not be fixed, but rather, as socially, historically, and culturally produced (Azzarito, 2010; Bordo, 2003). Using these approaches can allow us to expose and challenge the ways that bodies are produced through discourse, facilitating our resistance to dominant social structures (Barret, 2005; Gerbensky-Kerber, 2011; Numer & Gahagan, 2009). Important to feminist post-structural approaches is the work of Foucault, and in particular his conceptualization of power as relational, his ideas around disciplinary power, and the concept of evaluative gaze (Azzarito, 2009; Downing, 2008; Foucault, 1995).

The neo-liberal discourse of healthism and fitness has led to a dominant discourse that blames individuals for their choices in physical activity and nutrition and labels resultant issues, such as obesity, as a moral failing; this discourse is negotiated by girls and young women, draws attention to their bodies, and is added to complex gender discourses around femininity and slenderness (Azzarito, 2012; Gerbensky-Kerber, 2011; Mansfield & Rich, 2013; Rich & Evans, 2013). These discourses are normalized and perpetuated, tend to construct the body in reductionist ways, and results in the bodies of women being shaped by historical, political, and social trends, with those who do not fit the dominant discourse being silenced, marginalized, and oppressed (Azzarito, 2012, 2010, 2009; Azzarito & Sterling, 2010; Mansfield & Rich, 2013; Rich & Evans, 2005). Employing a feminist post-structural approach for this work offers an opportunity to explore societal level discourse in relation to adolescent girls’ and young women’s nutrition and physical activity.

### Study design: photovoice

Using a visual research methodology is appropriate for this work as discourses regarding healthism, weight, slenderness, and femininity are often perpetuated visually through media and popular culture (Azzarito, 2009). Azzarito et al. have used visual methodologies and feminist post-structural approaches to explore the construction of bodies in physical activity and physical education, for example, and suggest visual research can help girls and young women reposition bodies (Azzarito, 2012, 2009; Azzarito & Katzew, 2010; Azzarito & Sterling, 2010). Further, recent work suggests the need for health research to be more theoretical and embrace more creative and less typical methodologies (Rigg et al., 2014).

Photovoice, originally described by Wang and Burris (1997), is a specific visual methodology where participants take photos and actively engage in the research process (Wang & Burris, 1997; Wang et al., 1998). Photovoice is underpinned by theory such as education for critical consciousness, focusing on empowerment, feminist theory, and community or participatory approaches (Catalani & Minkler, 2010; Wang, 1999; Wang & Burris, 1997; Wang & Pies, 2004). Photovoice has three primary goals: to enable people of a community to record and reflect on strengths and concerns; to promote knowledge and critical dialogue through group consideration of photos; and to reach decision makers (Wang, 1999; Wang & Burris, 1997; Wang et al., 1998).

Photos offer a rich form of data, allow us to see the world as it is perceived by our participants, can teach us about social, political, and cultural conditions, and by allowing communities to represent issues, can facilitate making change (Wang & Burris, 1997; Wang et al., 1998). It has often been used to explore issues relating to the health of children and women (Brazg et al., 2011; Findholt et al., 2011; Shea et al., 2011; Vaughn et al., 2008; Wang, 2006, 1999). Other benefits of the photovoice methodology include promoting empathy, humanity, self-esteem, empowerment, and creativity (Royce et al., 2006; Strack et al., 2004; Wang, 2006).

### Population and recruitment

This project was approved by our institutional ethics review board, and closely followed the nine-step process outlined by Wang in her article describing the use of photovoice to mobilize change with youth (Wang, 2006). The first two steps involve recruiting a target audience and population. For this project, we worked with a local community advisory group on girls’ physical activity to be connected with potentially interested participants. We used purposeful sampling to select an organized group of potential participants who met regularly and focused on community development and issues relating to health. Once a potential group had been identified, the lead author visited their regular programming to get to know the participants, conduct recruitment, and leave information and informed consent packages. A group of seven girls and young women chose to take part, ranging in age from 13 to 26 (average: 19). The wide range in ages is due to the existing programming at the partner organization which emphasizes principles of youth engagement, leadership, and participation; as a result, youth programme participants and staff form a cohesive group across a range of ages. This sample is consistent with photovoice methodology which may use small sample sizes (Catalani & Minkler, 2010) and may include youth and young adults of varying ages (Wang, 2006).

Steps three and four of the photovoice process involve introducing the process and establishing informed consent (Wang, 2006). Following the initial recruitment meeting, a full-day workshop was organized to introduce the project and its goals, review ethical procedures, and the process for the project. Given the topic of girls’ and women’s bodies, and the political and personal nature of photos, best practices for conducting photovoice projects ethically were followed (Wang & Redwood-Jones, 2001). This included three types of consent: first, standard informed consent (and parental consent) was achieved by circulating information and consent packages to potential participants (and their parents, if below the age of 18 (n = 4 participants)) in advance for their review and written signature. All participants (and their parents, if applicable) were provided contact information for the lead researcher to ask questions or discuss in advance, and time was permitted at the beginning of the workshop for any final questions. Oral assent was also confirmed for all participants at the beginning of each session. Second, informed consent was sought from those who could be identified in photographs, by the participant photographers, following training at the first workshop. Finally, photographer consent was established at the end of the study, once the photos had been analysed and their potential meaning and implications discussed (Wang & Redwood-Jones, 2001). The ethical concerns of photovoice projects, photo-taking, storage, and submission were also discussed and practised at the workshop, and participants were provided with all necessary materials, and with a copy of their photos (Wang & Redwood-Jones, 2001).

### Procedures

Steps five through seven of the photovoice process involve the data collection procedures (Wang, 2006). The fifth step involves brainstorming ideas for photo-taking. This occurred in a focus group style discussion at the workshop where topics such as gender norms, trends, and stereotypes in girls’ and women’s health were discussed. Focus group discussions were audio recorded and transcribed verbatim for reference and presentation of direct quotes. The sixth step involves reviewing cameras and their use, which also occurred at the workshop. Participants were offered the option of using their own or borrowing a camera. Participants were also engaged in a photography skills-training session offered by a professional photographer (Catalani & Minkler, 2010; Strack et al., 2004). The final step for data collection involved providing the participants approximately 2 weeks to take photos. Photos were submitted electronically using our university’s secure file transfer system.

**Figure 1.**
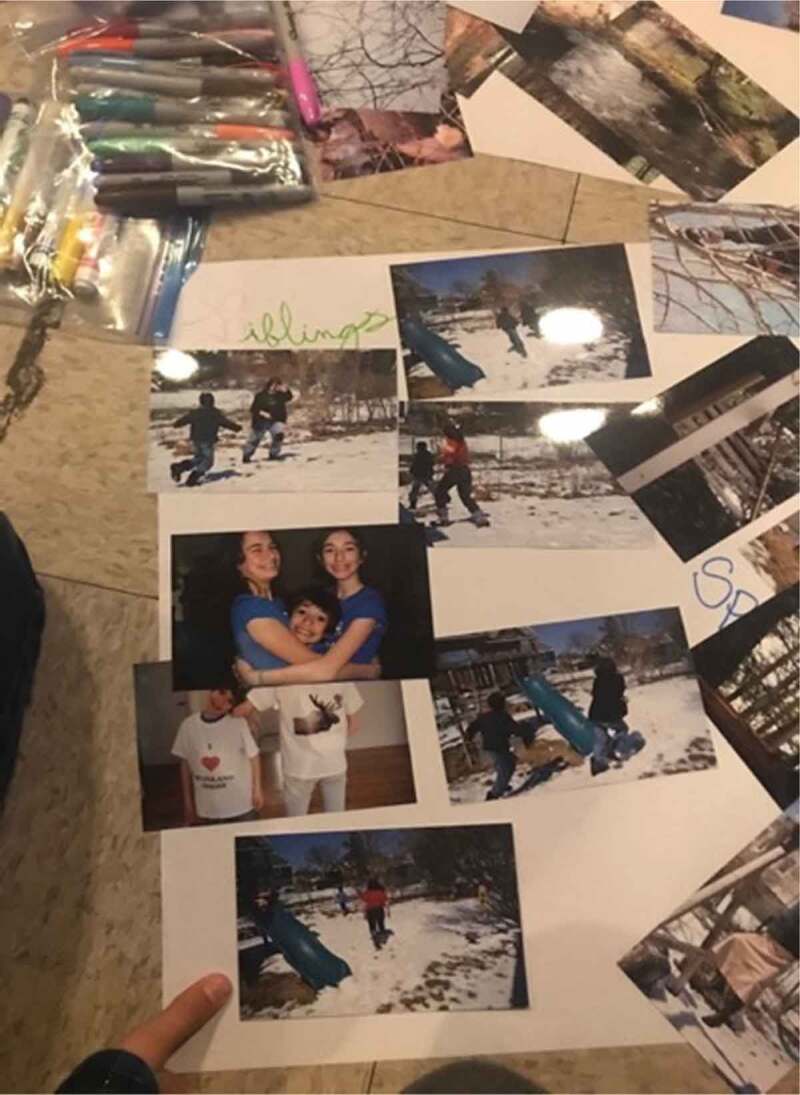
Images from the codifying process

**Figure 2.**
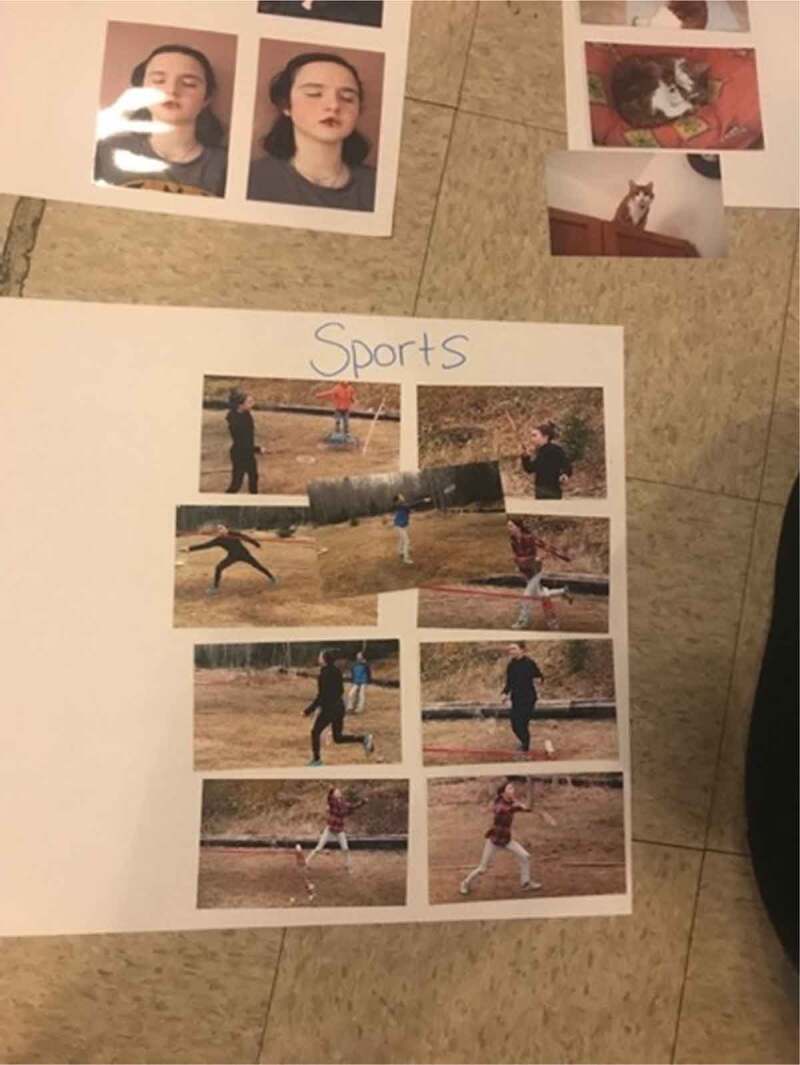
Additional images from the codifying process

The eighth step of the photovoice process involves data management and analysis (Wang, 2006). Photos that were received electronically were printed in advance of organizing a follow-up focus group style session for analysis. Each photo was printed twice, so that there would be a study copy and a participant copy, and all photos were numbered. The focus group analysis sessions followed the three-step photovoice process of selecting, contextualizing, and codifying (Wang, 2006; Wang & Burris, 1997; Wang et al., 1998). Selection involved each participant choosing a couple of photos that they thought to be meaningful or significant for further discussion. Contextualization involves analysing and giving context to those photos. This was accomplished using the SHOWeD method, which asks participants what they **s**ee, what they think is really **h**appening, how what is happening relates to **o**ur lives, **w**hy the issue/problem/strength exists, and what can be **d**one about it (Royce et al., 2006; Strack et al., 2004; Wang et al., 1998). Finally, codifying involved engaging the participants in a process of participatory thematic analysis, where their photos could be considered as a broader group to look for themes and trends (Wang et al., 1998). This process started simply by participants grouping similar photos together, and progressively gained depth. This process is visible in Figures 1 and 2. Steps five through eight were repeated a second time to permit further collection of photos and more in-depth analysis. Analysis sessions were audio recorded and transcribed verbatim as well.

**Figure 3.**
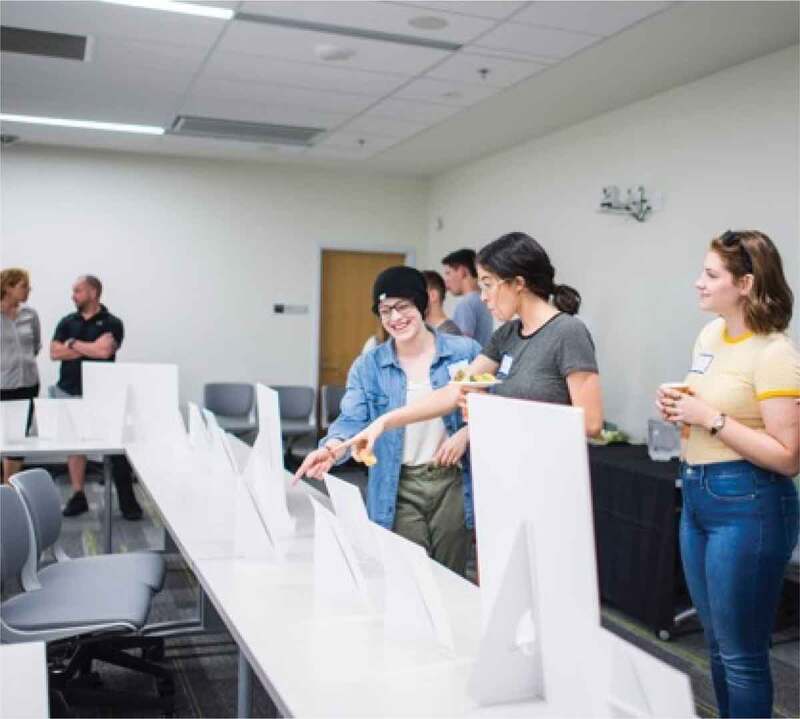
Images from the photo sharing gallery event

**Figure 4.**
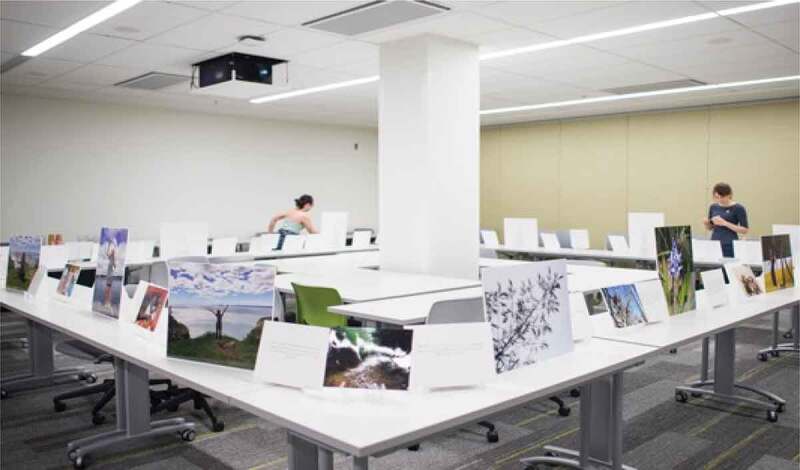
Additional images from the photo sharing gallery event

The ninth and final step in the photovoice process involves sharing photos (Wang, 2006). This was also conducted using a participatory approach whereby participants were asked how they wished to share their photos. They decided on a drop-in photo gallery style event at which we enlarged and printed their photos alongside some of the direct quotes that will be presented in the “Results” section. The gallery event was attended by the participants’ friends and families, the university community, and other stakeholders in girls’ and women’s health and wellness such as practitioners, recreation programmers, and representatives from local relevant government departments. The photographer from the original training workshop attended to take photos as well, with examples available as Figures 3 and 4.

## Results

Using the codifying process, five themes were developed with the participants. Upon further analysis, these are presented below as three themes and two subthemes. The first theme, *(Breaking) Stereotypes*, includes a subtheme of *Conflict and Contradictions*. The second theme, *Emotional Safety*, includes a subtheme of *Practice, Confidence, and Pride*. The final, *Being Outside in Nature*, is presented primarily visually.

### Theme 1: (breaking) stereotypes

**Figure 5.**
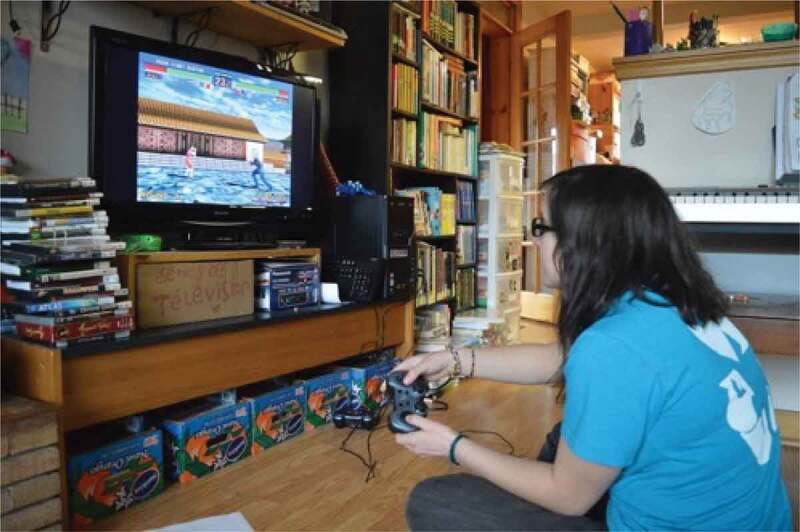
Example of girls challenging norms

**Figure 6.**
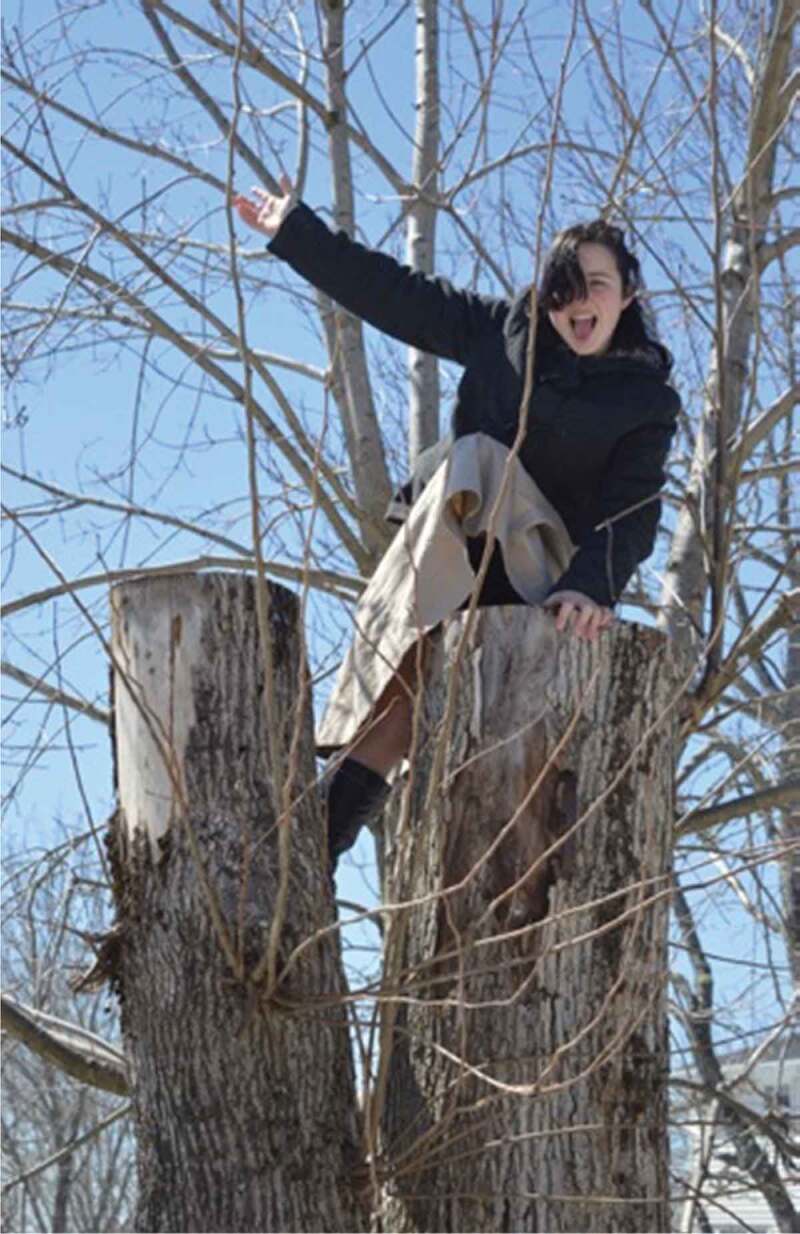
Additional example of girls challenging norms

The first theme of *(Breaking) Stereotypes* was the over-arching theme from this work. Participants discussed how “everything is gendered*”*; they noted the existence of common gender norms and roles and recognized that these are perpetuated through institutions and environments. They connected gender norms to health, discussing stereotypes around sports and dieting, and acknowledged their historical production and perpetuation. The girls and young women also frequently discussed challenging these norms and stereotypes, by doing what they might not be “supposed*”* to do, or what might be considered unexpected. One said, for example, “I always felt that I was not necessarily interested in those kinds of I don’t know, certain games or Barbie dolls […] I always was the outdoor type […] on my bicycle, or in the water, like you know […] not worrying about my make-up, not worrying about my hair*”*. The participants also frequently took photos of this nature, such as playing video games or climbing a tree while wearing a skirt, as pictured in Figures 5 and 6.

The participants also discussed situations in which they do not feel as safe challenging gender stereotypes, which is the reason for the brackets around the word “breaking” in the theme label. One girl said, for example, “I loved to play soccer […] but I never knew how to approach a group of boys who were playing […] I would just kind of stand there and watch them and they would never invite me*”*. The participants noted that due to social, generational, and historical influences they sometimes contributed to the perpetuation of norms, and also discussed examples where they engaged in what might be considered stereotypical behaviour that they enjoyed doing. These complexities were often connected to appearance and body ideals. One girl said, for example, “everybody at one point like definitely looked in the mirror and been like ‘oh I wish that I took just one size smaller”; the girls recognized the unrealistic nature of these ideals, but also how entrenched and difficult to challenge they can be.

These ideas were often connected to media, and both physical activity and nutrition. The participants noted media, especially social media, as a primary source for gender norms and body ideals, and connected this to the dieting and food industries, with one saying, “like on Instagram […] there’s always pictures and it’s like ‘try this tea’ or whatever, it’s like always like this girl in a bikini who looks unrealistically tanned and flawless and stuff*”*. Similarly, another participant noted, “there aren’t that many ads that show girls and physical contact sports*”*, showing how gender norms and stereotypes are perpetuated in media’s portrayal of athletes.

#### Subtheme: conflict and contradictions

**Figure 7.**
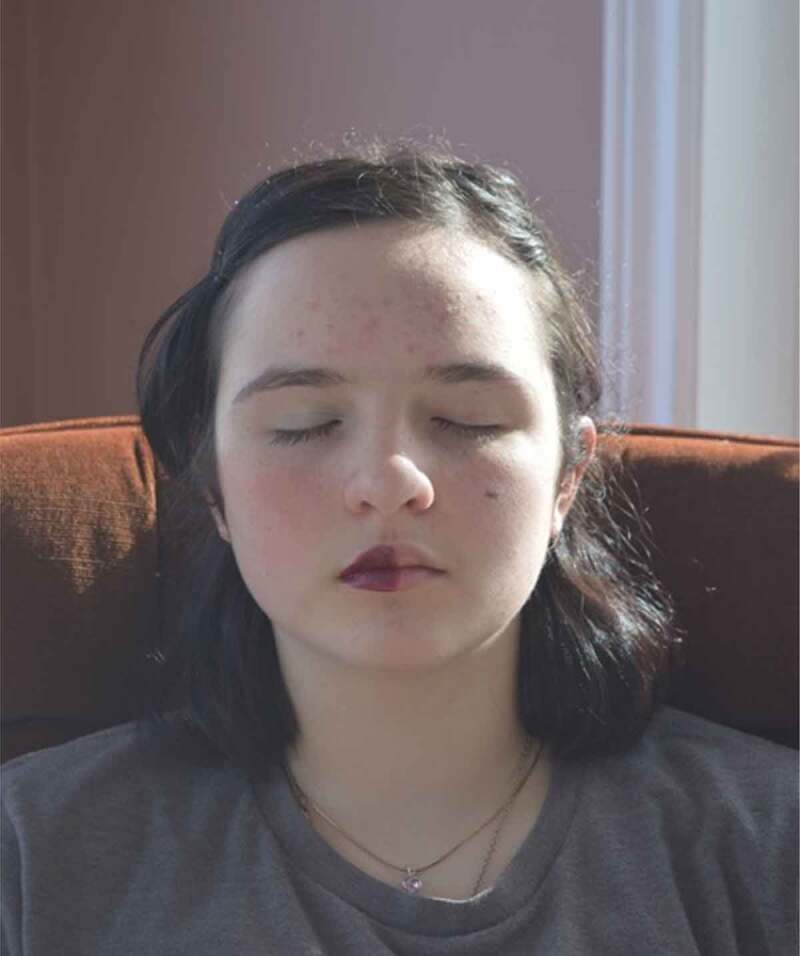
A girl with makeup on only one half of her face

The girls and young women in this study discussed their regular confrontation of gender norms and how this relates to a variety of conflicts and contradictions. As alluded to above, the participants discussed how it can be difficult to challenge norms; One said, “it’s very hard sometimes because you don’t want to be a stereotype girl, but sometimes some girls like stereotypical things*”*, showing how they do not want to be seen as stereotypical, and may avoid behaviours they enjoy to avoid that perception. They also connected this conflict to clothing and makeup, with one girl saying,

I worry sometimes, I’m like, ‘why is this connected to my happiness?’ that I want to put on make-up […] I worry like am I doing this because I want to? I feel like I am, but then I’m also like maybe I just want to because … society.

which is a conflict pictured in Figure 7 by a girl wearing makeup on only one half of her face.

**Figure 8.**
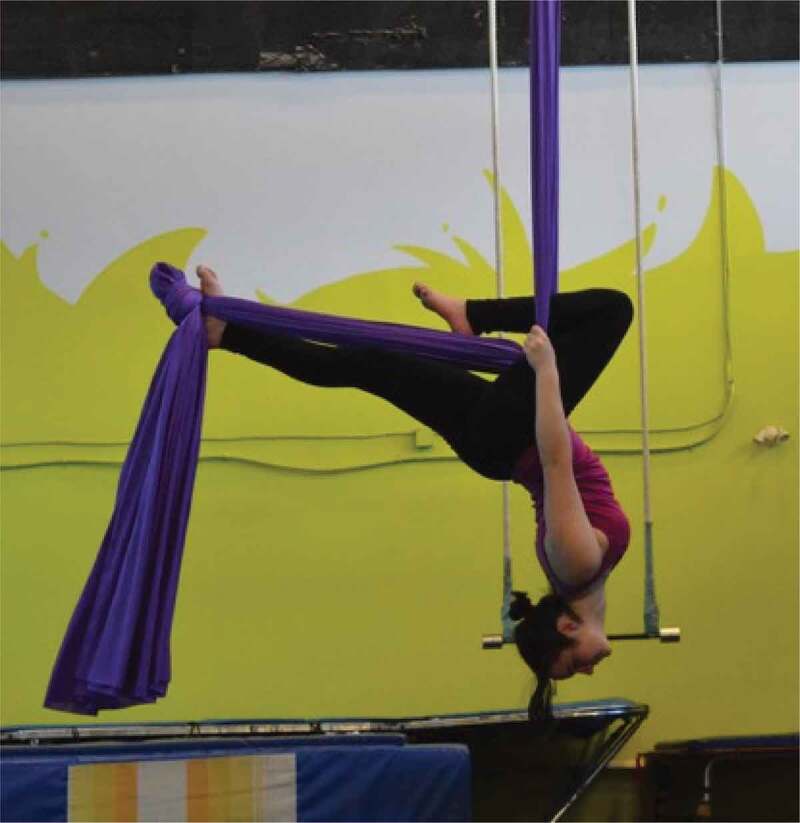
Example of contrasting gender performances

**Figure 9.**
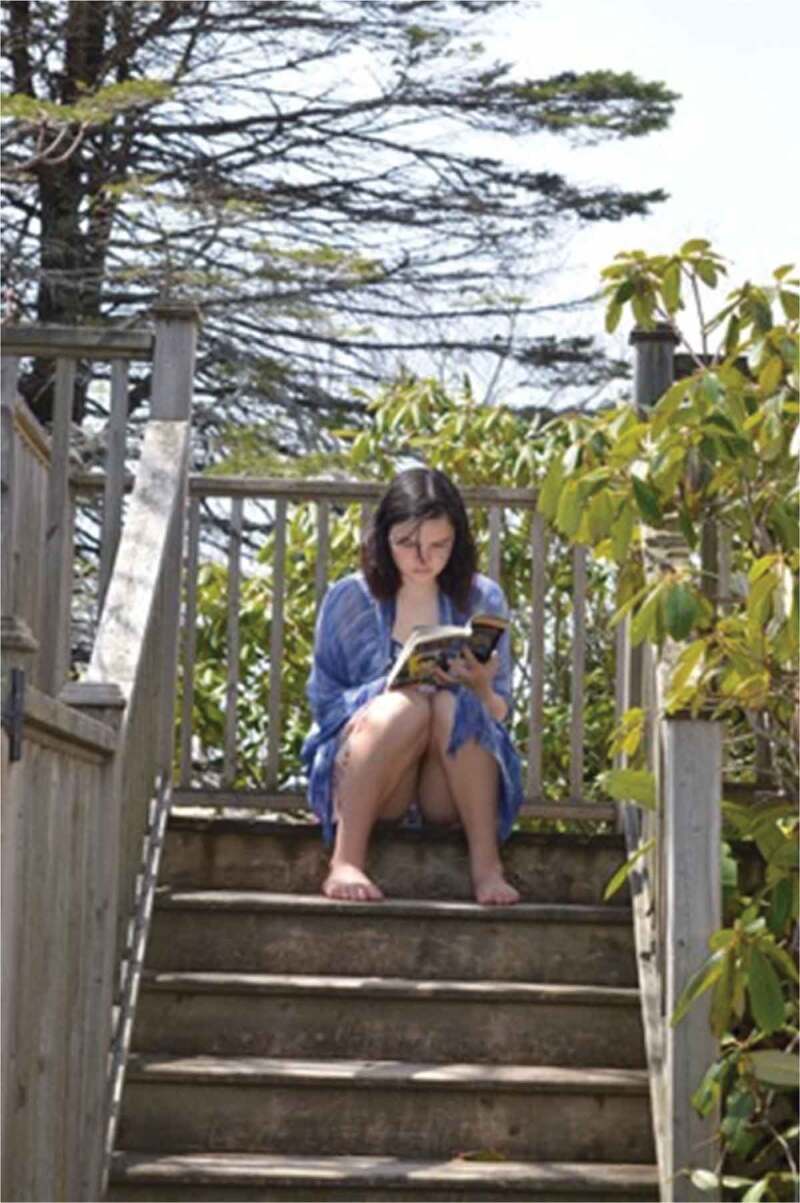
Example of contrasting gender performances

Well-aligned with the feminist post-structural theory guiding this work, the participants described gender as a performance, with one saying, “it’s almost like you had to act in a certain way, it’s like your performance*”*, describing the societal expectations for their actions and behaviours. The participants also discussed their own performances as contradictory, depicted in Figures 8 and 9, which show the same girl doing something athletic that might be considered brave and unexpected alongside her reading quietly. Relatedly, one girl said, “we don’t like people fussing over like the one little thing that changed […] So if one day you felt like wearing makeup, and you came out with makeup on, everyone would have a reaction”, further connecting the idea of conflicts and performances to attracting attention regarding appearance.

The participants also connected these conflicts and contradictions to nutrition and physical activity. Regarding nutrition, one girl said, “if you take a really small portion […] it’s, ‘oh you haven’t eaten much, you must be on a diet’. Or you eat like a really big portion, and ‘wow that was a lot of food’*”*, suggesting how it seems there is no right amount of food a young woman can consume without facing criticism. Another girl said,

when we talk about men’s eating habits it always comes back to strength, so it’s always like ‘oh he’s a growing boy, he needs to eat more’. … but […] we don’t talk about girls’ health […] we always talk about appearance

describing a significant gender disparity in expectations around nutrition. Contradictions were further noted in physical activity. One young woman said, “like in volleyball or whatever, the girls, like part of their uniform is to wear, like really short shorts and the boys it’s not”, noting double-standards in dress codes that suggest they should cover their bodies in class but not so during physical activity.

### Theme 2: emotional safety

**Figure 10.**
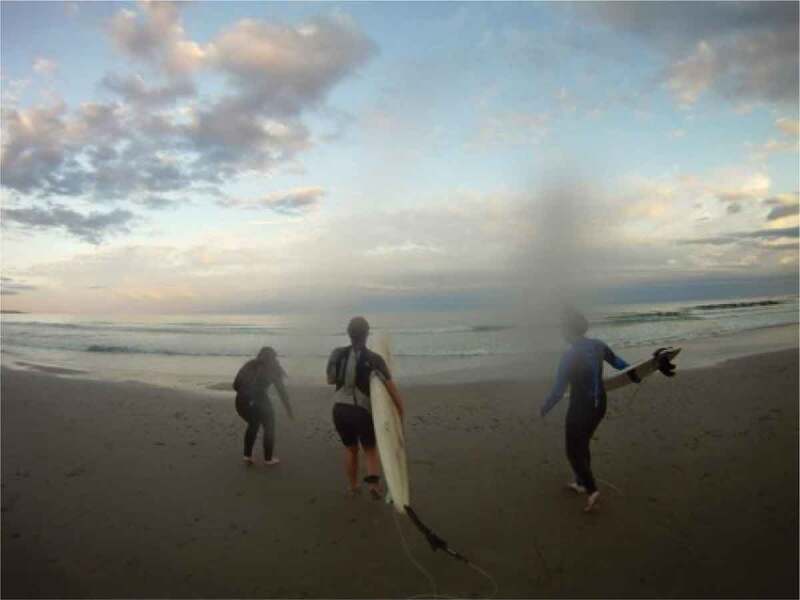
An example of friends providing emotional safety

**Figure 11.**
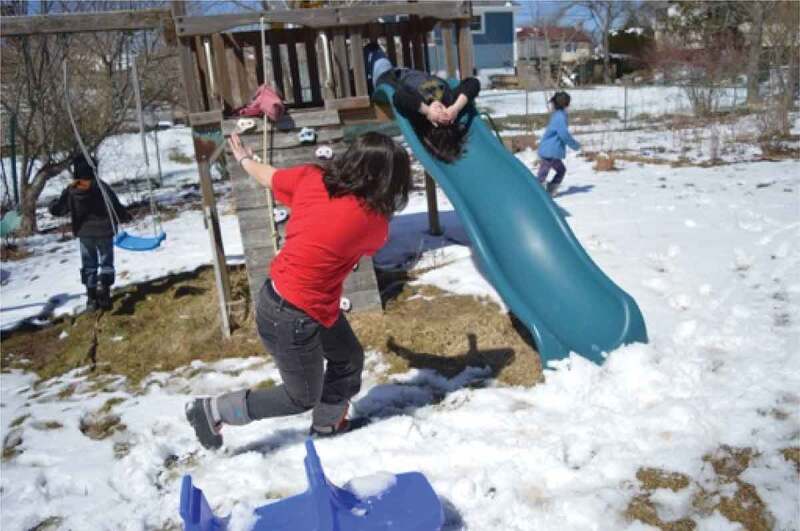
An example of family as emotional safety

**Figure 12.**
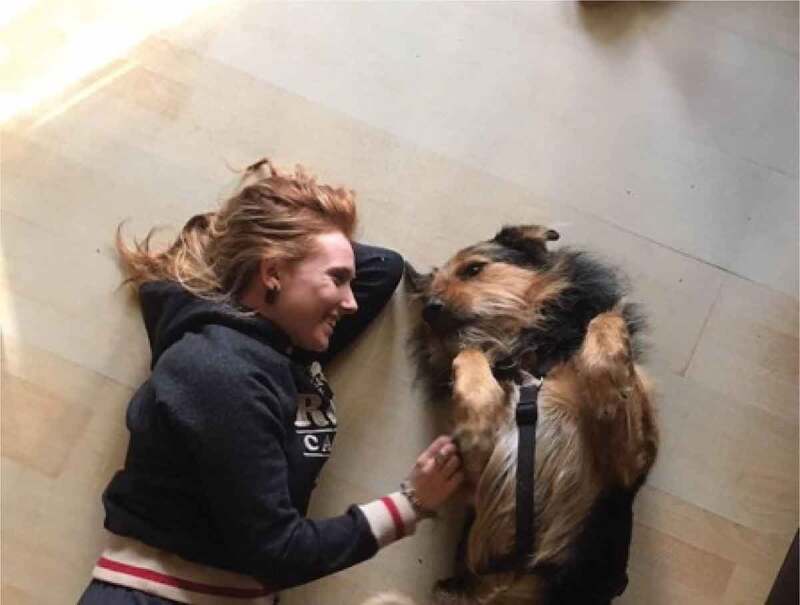
An example of pets as emotional safety

The term emotional safety is one typically used in the field of psychology regarding couples’ relationships (Catherall, 2006); however, it was adopted by our participants to name this theme. Here, they applied the term more broadly to describe the circumstances and facilitators that made them feel comfortable, safe, loved, appreciated, protected, expressive, assured, and confident. Having emotional safety supported the girls’ and young women’s resilience in order to be able to navigate the previously discussed gender stereotypes and norms, and the associated conflicts and contradictions. As part of this theme, the participants frequently discussed photographs of their friends, families, and pets. Friends, family, and pets provided social and emotional support to our participants, and they discussed the importance of trust and feeling comfortable around one another. Describing Figure 10, for example, one participant said, “these are two of my best friends and they both live away and this is like a really special memory […] one of our favorite pastimes when we were in high school was all going surfing together*”*. Similarly, in referencing Figures 11 and 12, family and pets were also discussed as facilitators of emotional support, and often, encouragers of physical activity.

The girls and young women in this study also discussed things that challenged their emotional safety. Though most participants were quite young, they all discussed their physical safety as relating to gender and health, and many described experiences where they had been made to feel uncomfortable. The girls and young women frequently made statements like, “we feel like we have to be more careful when we’re alone*”*, demonstrating their perception that women are often exposed to harassment and danger. While these examples were rarely overtly connected to physical activity or nutrition by the participants, it’s not difficult to see the possible connections. As girls and young women feel challenged by travelling alone, simply getting to or from opportunities, or pursuing independent forms of activity like walking or hiking could be limited. Additionally, they perceived an absence of women as coaches and mentors, so it is understandable that girls and young women may lack emotional safety and support in many contexts where physical activity might typically be encouraged.

#### Subtheme: practice, confidence, and pride

**Figure 13.**
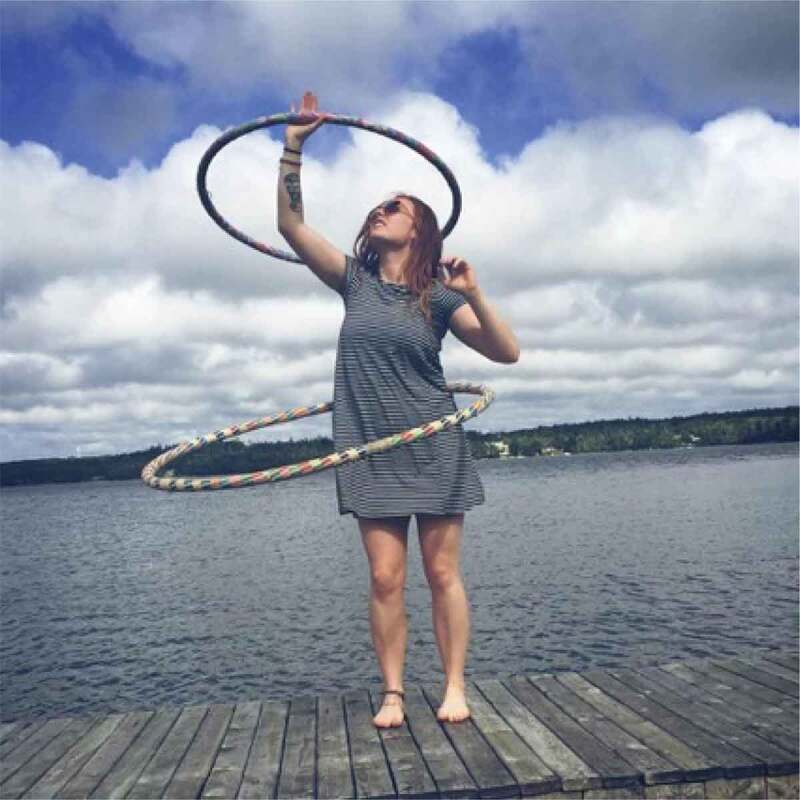
Example of practice, confidence, and pride

**Figure 14.**
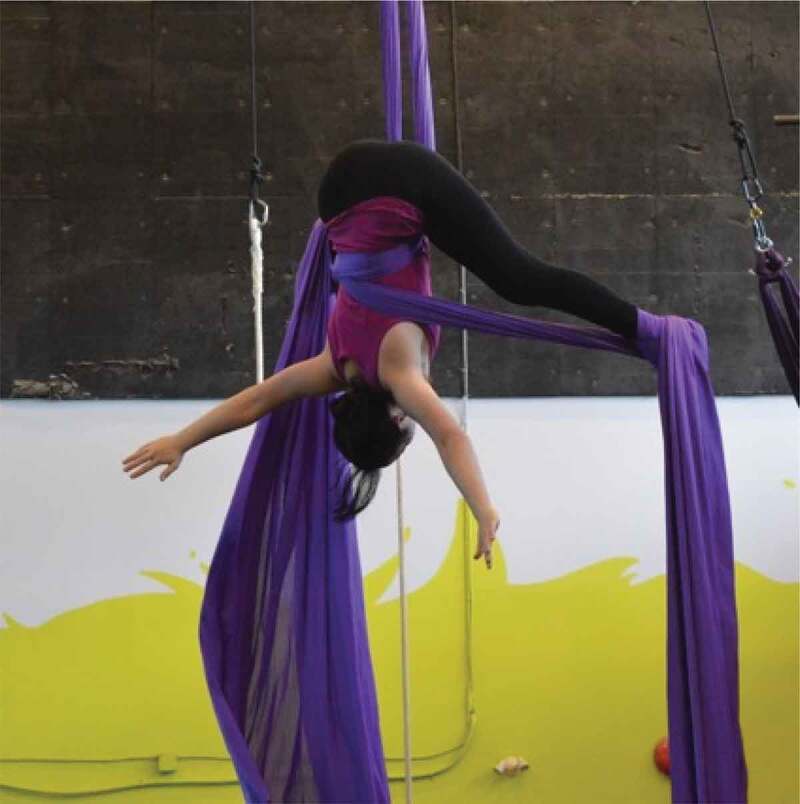
Additional example of practice, confidence, and pride

**Figure 15.**
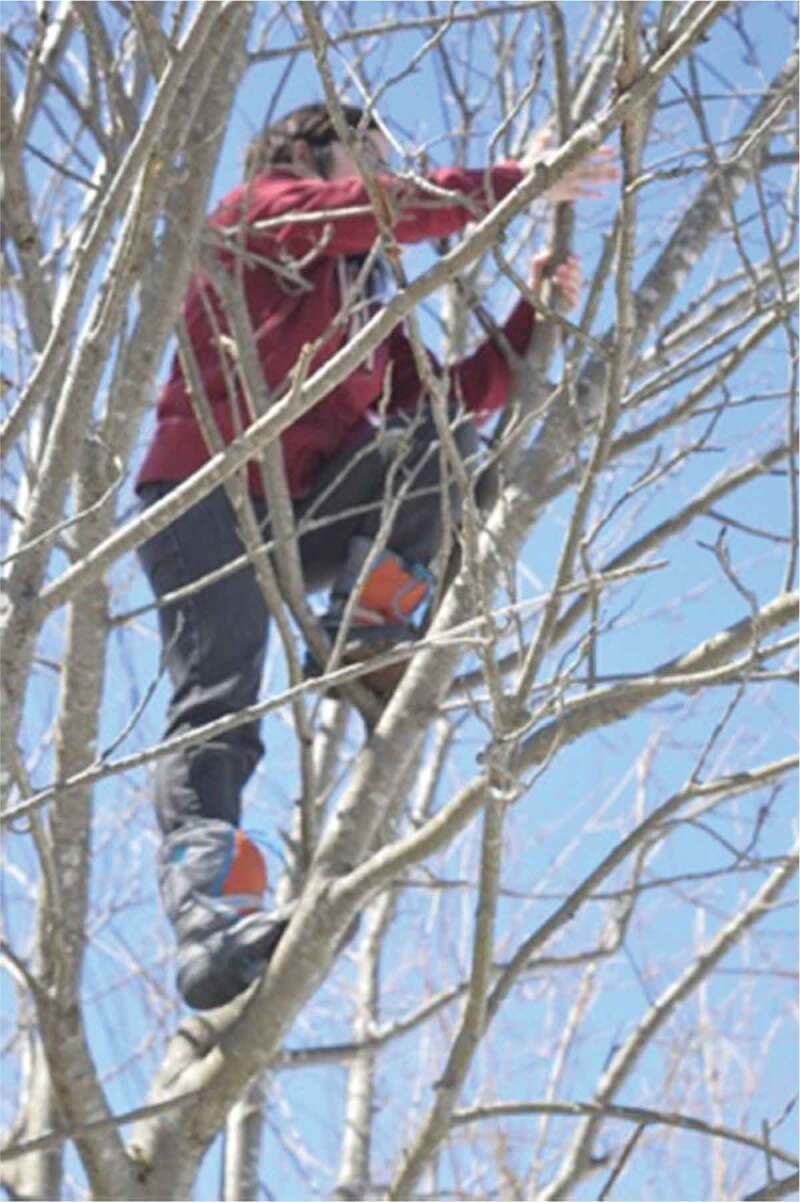
Further example of practice, confidence, and pride

**Figure 16.**
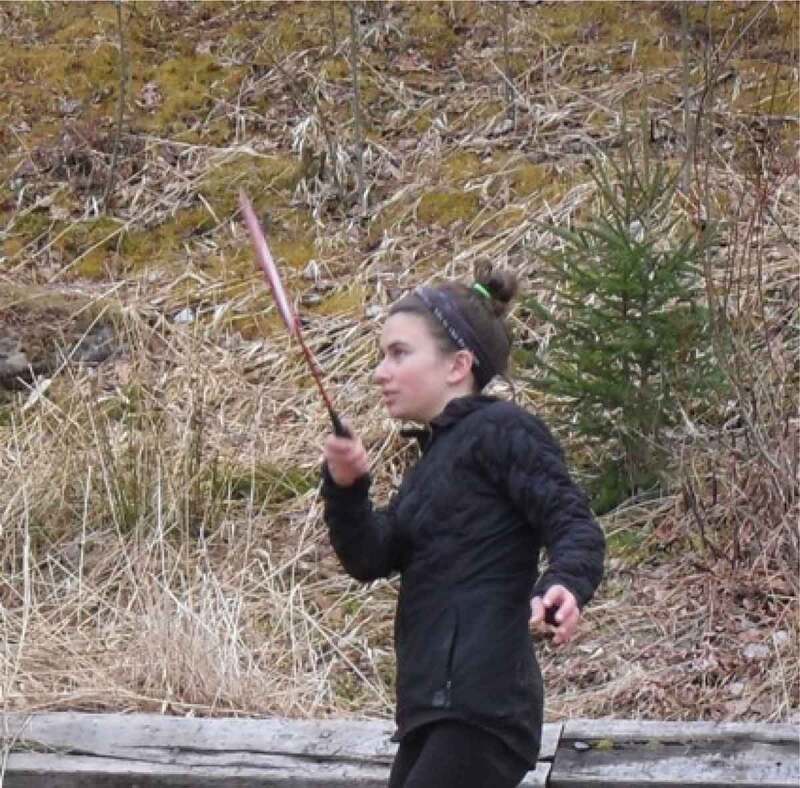
Another example of practice, confidence, and pride

The girls and young women in this study frequently discussed and photographed activities that required practice and illustrated moments of confidence and pride. Regarding the images in Figures 13–16, for example, one girl said, “It’s cool that these photos are all of us […] being alone, but we look fearless”, while another agreed, “just proving that we can do things and not just sit around”. It seemed that developing a sense of familiarity and building confidence allowed the girls to overcome fears, challenges, and gender role contradictions. This was most frequently connected to physical activity and active accomplishments and examples where the participants felt proud. It seems, in moments when girls and young women feel emotionally safe, they are able to better navigate some of the previously discussed gender norms and potentially challenge or subvert the dominant gender discourse.

### Visual theme: being outside in nature

**Figure 17.**
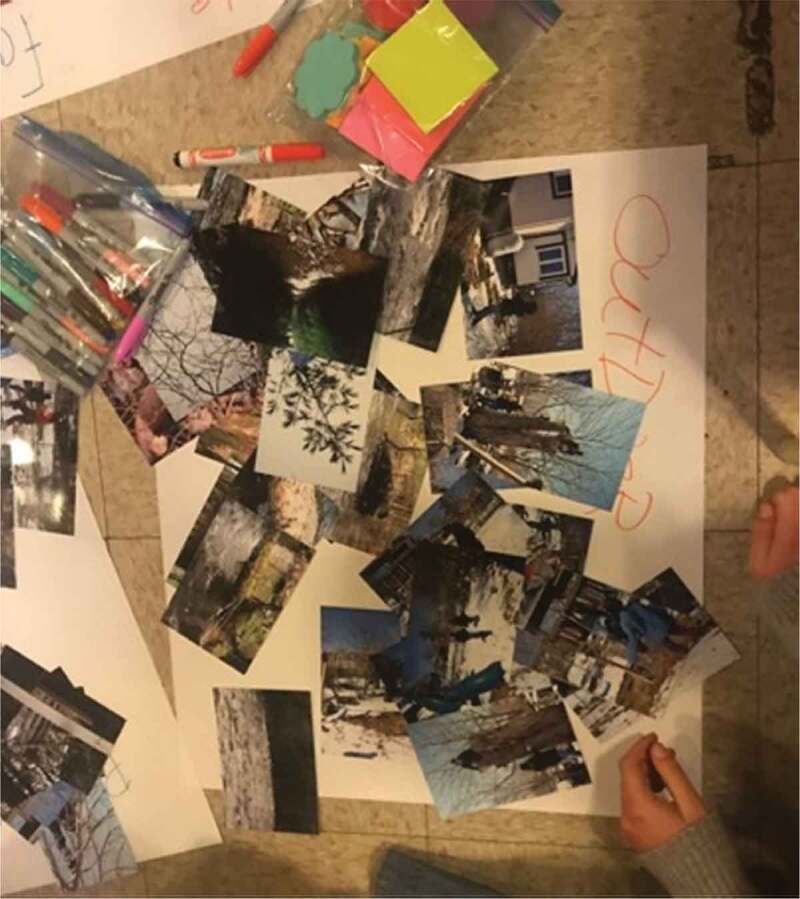
A growing pile of photos labelled “outdoors”

**Figure 18.**
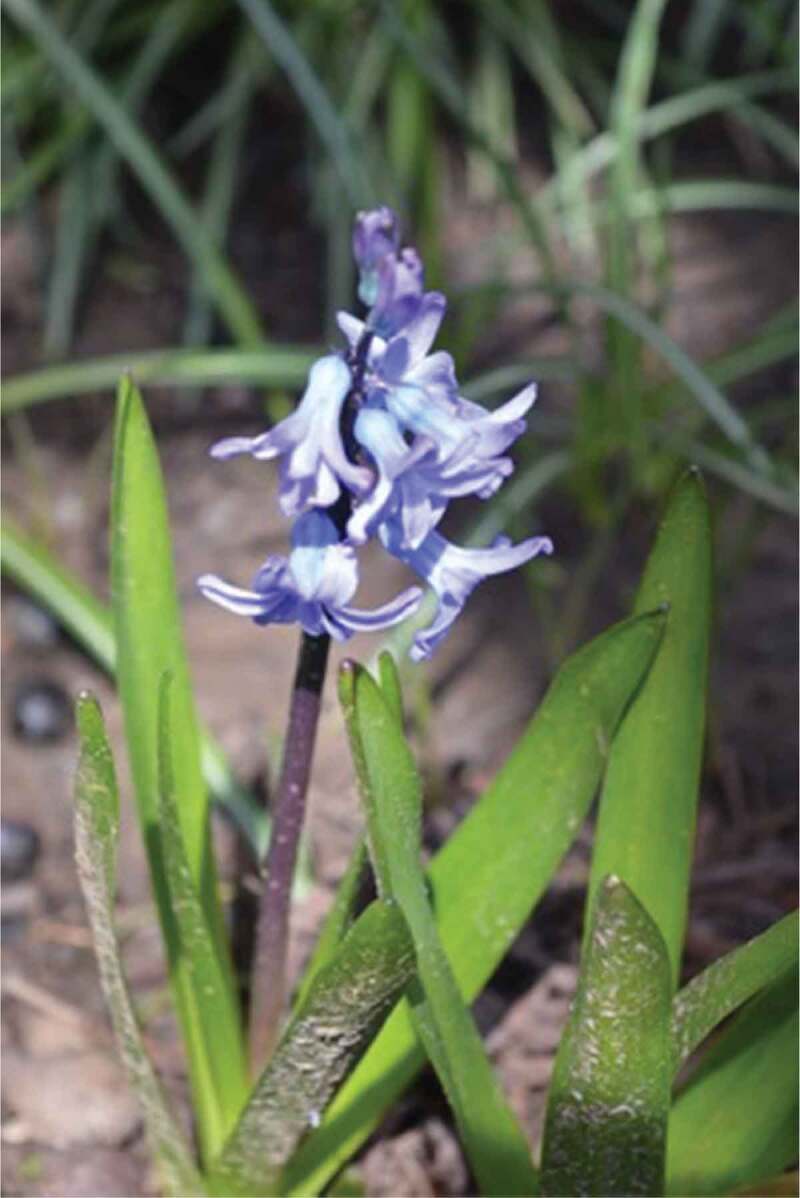
Example of natural elements photographed

**Figure 19.**
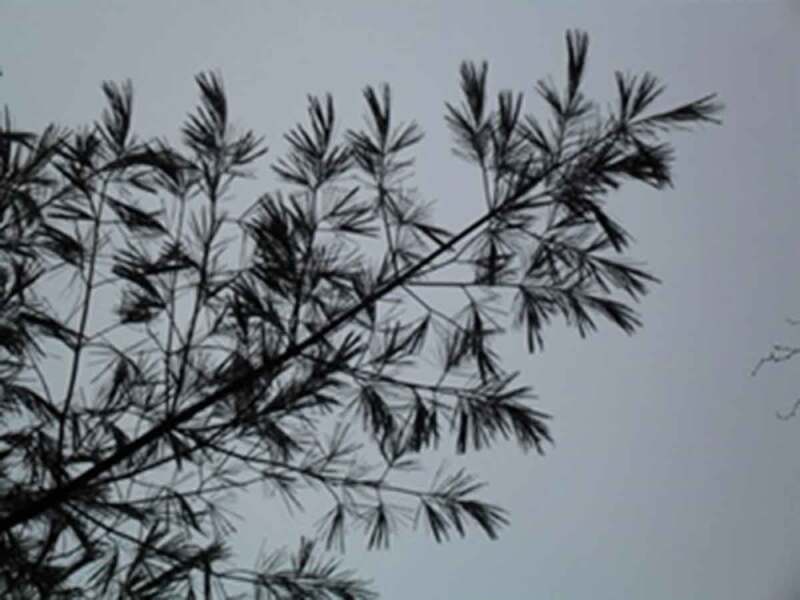
Additional example of natural elements photographed

**Figure 20.**
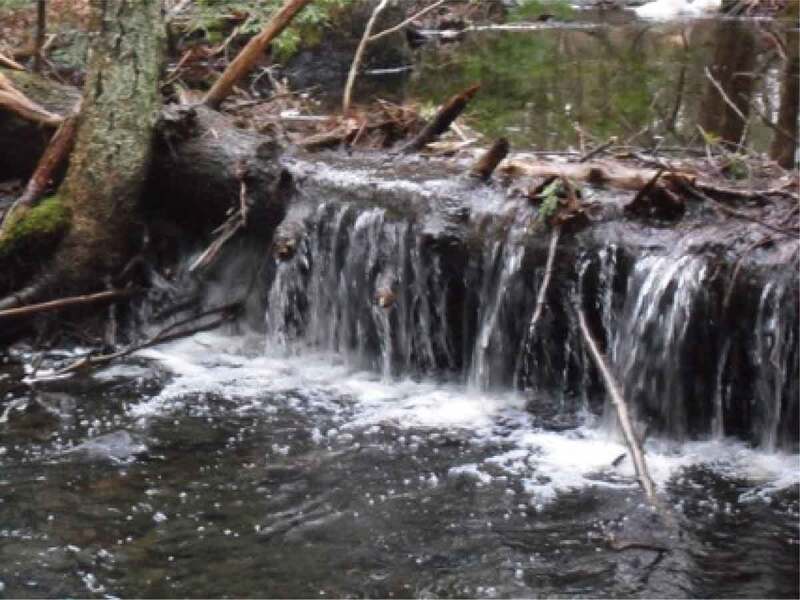
Further example of natural elements photographed

**Figure 21.**
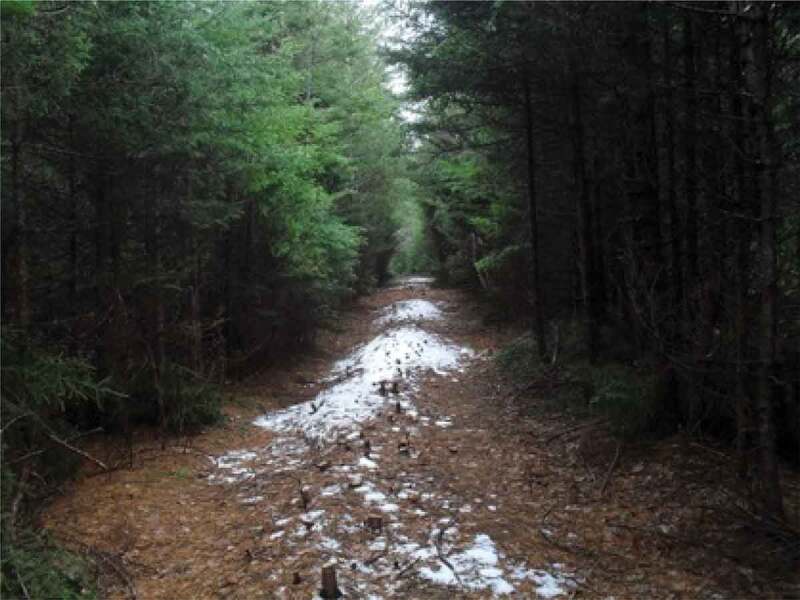
Another example of natural elements photographed

The final theme that was developed with the girls and young women is *Being Outside in Nature*. Interestingly, the importance of being outside was only occasionally overtly discussed by the participants. One said, for example, “growing up I loved to play soccer and I don’t think it was about the soccer, I think it was about me wanting to be outside and wanting to be active*”*. This importance of being outside, was something that appeared visually, through the photos, as shown in Figure 17, through a growing pile of “outdoor” photos being grouped by the participants. This is also evidenced by the photos that have already been presented, many of which were taken outside; as such, this theme is presented primarily visually. In many cases, participants took photos of natural elements and selected them for discussion. A participant discussed the photo in Figure 18, saying, “our yard outside is really gorgeous because there are always, you know, there’s some sort of flowering bloom*”*. Similarly, a participant described the photo in Figure 19, saying, “I like the […] contrast, there’s a grey sky and the dark branches*”*. Other images that depict nature are visible in Figures 20 and 21. While these images were not always overtly connected to gender or health, they held significance for the participants, and illustrate the importance of connecting with nature to the girls and young women.

**Figure 22.**
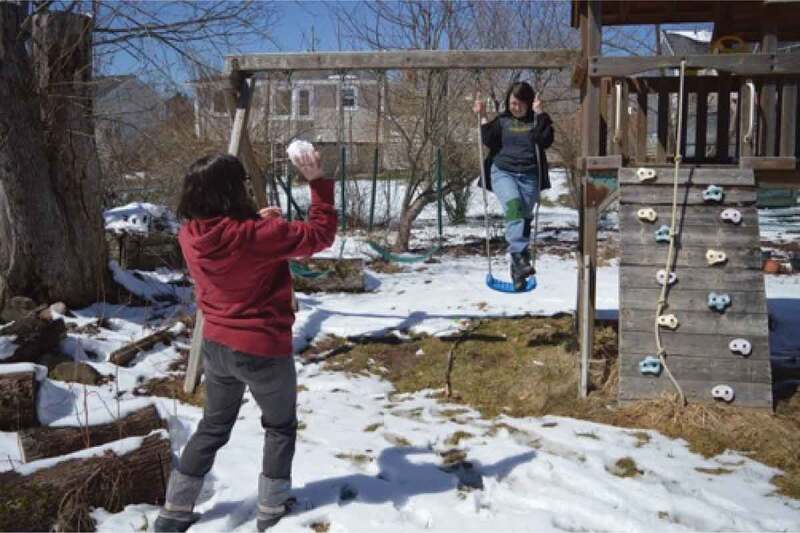
Connecting nature and physical activity

**Figure 23.**
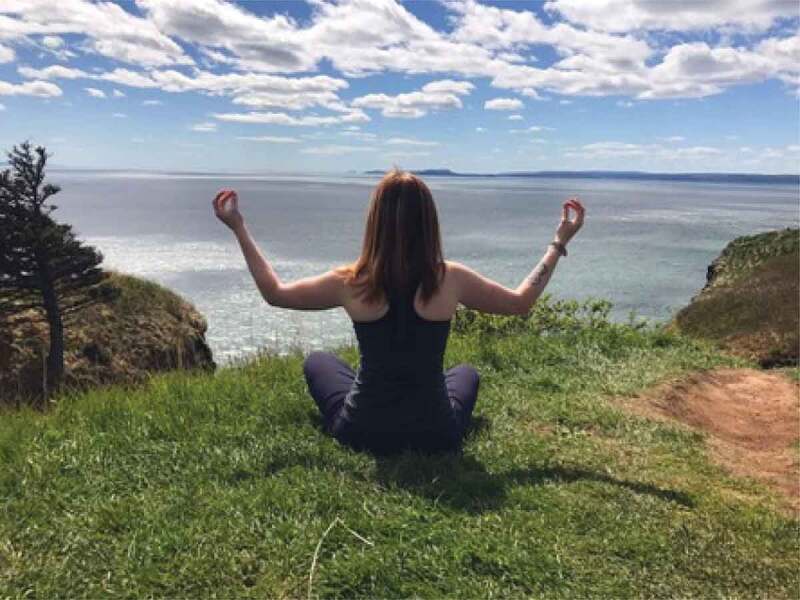
Connecting nature and physical activity

In several other cases, the participants connected being outside in nature directly to their health, particularly regarding physical activity. Describing the photo in Figure 22, a young woman said, “yah it’s just […] being active […] throwing snowballs at each other […]

just being outside”. Finally, some participants also drew connections between nature, physical activity, and confidence. About the photo in Figure 23, one said, “it’s like a long hiking trail and I had like just finished the hike […] so it was like really, really long […] it was a really pretty view*”*. These quotations highlight how being outside often acts as the context in which girls are able to find emotional safety, such that they can negotiate complex gender stereotypes, conflicts, and contradictions, and engage in healthy behaviours.

## Discussion

Discussions with the girls and young women who took part in this project revealed how they conceptualized gender norms, and associated complexities, conflict, and contradictions. They acknowledged traditional norms and stereotypes and ideas regarding what they were supposed to do or how they might be expected to act. This understanding of norms is aligned with photovoice’s theoretical underpinnings related to critical consciousness, where through critical examination of society and a process of self-awareness, advocacy for change can take place (Freire, 2000; Wang & Burris, 1997). This understanding or self-awareness is further aligned with feminist post-structural thought because it can be interpreted as awareness of social forces or power relations.

From a feminist post-structural lens, we consider analyses of power relations and the production of knowledge. Foucault questions history and knowledge, suggesting they depend on social context and therefore cannot and should not be separated from power relations (Downing, 2008; Felluga, 2015; Foucault, 1995). Power, or rather, the effects of power, then play a role in how bodies are controlled, such that we go about our daily actions, our performances, generally, trying to not be disruptive of norms. Foucault’s discussion of the panopticon suggests that, like in a prison with a central tower for surveillance, we have an impersonal or disembodied relationship with power, and a constant sense of evaluative gaze (Azzarito, 2009; Downing, 2008; Duncan, 1994; Foucault, 1995). Acts outside of the dominant discourse are then questioned or challenged, which was noted by the participants in this project.

The girls and young women in this project took pride in challenging norms and doing what might be unexpected of them. They enjoyed getting messy, being active, playing outside, not worrying about their physical appearance, and embraces the complexities associated with subverting the dominant discourse. That said, the girls and young women also discussed examples of their own conformation or perpetuation of norms, and situations in which it is not safe of comfortable to break norms, particularly regarding physical activity. Dominant discourse can only achieve dominance in comparison to an alternative. It is not problematic to engage in what might be considered feminine; it is oppressive when the behaviour is questioned or when power relations dictate their control. The girls in this project sometimes chose that which is feminine. This awareness of subjectivities illustrates the agency of these young women.

Women consistently face regulatory discourses regarding their bodies (Azzarito, 2009; Bordo, 2003; Gerbensky-Kerber, 2011; Mansfield & Rich, 2013; Rich & Evans, 2013). Based on negotiations of discourses and power relations, they construct meanings of “normal” and “good” bodies, and comparatively, “bad” or “deviant” ones; we therefore experience body discipline as a form of social control (Azzarito, 2010; Bordo, 2003; Duncan, 1994; Gerbensky-Kerber, 2011; Mansfield & Rich, 2013; Wright et al., 2006). The girls in this study recognized those societal pressures and how certain bodies are privileged in certain contexts and connected this to discourse about dieting and weight management, and media, where ideal bodies tend to be presented and reproduced. This is also aligned with photovoice work that has demonstrated girls and young women recognize the health of their bodies not only in a physical, biomedical sense, but in a more holistic and social sense as well (Shea et al., 2011).

The participants in this study also critically discussed confronting gender norms daily, and the ongoing internal conflict associated with not wanting to appear as a stereotype by taking on traditional gender roles. This conflict is a result of norms, ideals, and the requirement to engage in competing discourses, and the resultant repercussions. They connected these conflicts directly to health, through physical activity and nutrition, in the above-discussed examples of taking no appropriate amount of food, and double standards faced in physical activity attire. The participants recognized the gendered nature of these discourses, and their bombardment by contradictory messaging in media regarding body ideals and from food and dieting industries. These examples highlight the discourse focused on the bodies of girls and women, how the relational effects of power serve to control bodies through surveillance, evaluation, and all illustrate how the bodies of girls contend with political, social, historical relations (Azzarito, 2009; Butler, 1993; Downing, 2008; Duncan, 1994; Foucault, 1995).

The theme of *Emotional Safety* highlights the importance of social influence for issues relating to health of women and girls, gender, body image, physical activity, and nutrition, which is consistent with previous literature (Carey et al., 2014; Eisenberg & Neumark-Sztainer, 2010; Fitzgerald et al., 2012; Mackey & Greca, 2007; Rancourt et al., 2014; Slater & Tiggemann, 2011; Standiford, 2013; Witmer et al., 2011). The girls also revealed the importance of practice and developing familiarity, and opportunities for creativity, exploration, and self-expression. While they used the term “self-discovery”, a feminist post-structural analysis might suggest, rather than discovering, participants are exploring available subject positions. In many cases, girls have to choose between feminine discourse and pursuits where they might be perceived as active or strong. Emotional safety helped them to navigate these conflicts.

Finally, while *Being Outside in Nature* was not discussed in as much length as the other themes, it emerged as significant and meaningful through photos. The connection between nature and health, particularly regarding climate change, is an emerging area of focus for health promotion (Hancock, 2017, 2015). While the participants in this study demonstrated an important connection with nature, previously literature is conflicted. Nature and outdoor pursuits have historically been a male-dominated space, though outdoor adventure programming has been associated with increased physical activity in adolescent girls, as well as resilience and mental health benefits (Boniface, 2007; Cleland, 2005; Massa, 2015; Overholt & Ewert, 2015; REI, 2017). Other research has noted the importance of women engaging with the outdoors for empowerment, helping girls challenge assumptions, conventional femininity, and beauty ideals (Massa, 2015; Whittington, 2006), and has used photovoice to explore girls’ outdoor physical activity (Sackett et al., 2018). Additional literature points to feelings of fear and vulnerability outside (Boniface, 2007; Dooley, 2016; McNiel et al., 2012; REI, 2017; Wesely & Gaarder, 2004). In this study, images of nature connected the themes of this project together, with many images of outdoor pursuits representing emotional safety, confidence, and challenging norms. More research is warranted to further explore girls’ and women’s connection with nature as a context for health promotion and its potential for helping address gender stereotypes.

## Strengths and limitations

This project’s primary strength is the use of the photovoice methodology. Photos provide rich data and the opportunity to engage with participants beyond traditional research methodologies. Credibility is facilitated by the participatory nature of this work, and the relevance to the involved community facilitates authenticity and trustworthiness of the data. The research also addressed previously identified gaps in the literature by involving older adolescent girls and using a comprehensive approach that considers physical activity and nutrition as clustered health behaviours (Spencer et al., 2015). Finally, using a health promotion lens in combination with feminist post-structural theory and the photovoice methodology is a novel application of research methods.

In terms of limitations, photovoice presents logistical challenges in recruitment, coordinating meetings and collecting and managing photo data. These were mitigated to the best of our ability by working with an existing group of girls from a community organization and developing systems for photo submission and organization. The data presented in this project may also be limited by several participant characteristics, including the wide age range and the fact that there was a group of three siblings among the participants (non-siblings might have shared a wider variety of perspectives). As well, the participants in this study were limited by lacking racial and ethnic diversity as the majority of participants were White. While the focus of this project was on gender, we acknowledge that health is further complicated by race, and future research should aim to explore these topics using a more intersectional lens. Three participants also had been or were being homeschooled, and as such might have carried different perspectives than traditional public-school youth; however, this may have also acted as a strength as they were able to bring a critical lens and often unexplored perspective. Finally, the community organization from which the participants were recruited has outdoor programming as a focus, which may have resulted in volunteer bias and may explain the emphasis of our participants on the connection.

## Implications

We believe there are several important implications of this work. First, as noted above, photovoice, in combination with feminist post-structural theory offered an effective method to conduct participatory research in the area of girls’ and young women’s health and well-being. We would recommend others adopt both this and other forms of creative and visual methodologies when working with this population to explore challenging issues like gender norms. As well, practitioners might adopt similar methodologies in their practice with girls and young women to create space for critical reflection through creativity, especially in adopting participatory approaches. In this study, we found that girls and young women recognize and are well-versed in gender norms, stereotypes, and discourses related to gender and health. They are mindful of their own implicitness, perpetuation, and challenging of these norms and discourses; practitioners should encourage this self-awareness and mindfulness by encouraging both the deliberate adoption and rejection of stereotypes and norms, accompanied with discussion, reflection, and critical consideration. Important in this study was social support of peers and family members, especially in opportunities for girls and young women to engage in self-expression and creativity. We suggest that practitioners help create space for girls and young women to connect with one another in these ways. Finally, the importance of connecting with nature for the well-being for women and girls is an interesting and novel finding of this work that may contradict previous research. We suggest future research be conducted in this area to explore the sense of connection girls and young women may have with nature regarding their health and well-being. We also suggest practitioners not assume girls and young women to be disinterested in outdoor pursuits, and to encourage girls and young women to explore the connection between planetary health and their own well-being.

## Conclusions

The participants’ recognition of and reflections on gender norms and stereotypes connects to both feminist post-structuralism and photovoice’s theoretical underpinnings. Breaking or conforming to norms, disrupting discourse, and challenging expectations, connects to concepts like relational power and evaluative gaze. Girls in many cases embrace subverting the dominant discourse, but in others are more challenged. This connects to the construction and production of their bodies and the regulatory discourses of thinness. The girls and young women in this study also recognized the relational nature of power, and how some bodies are privileged in certain contexts. Regarding the contradictions faced, participants acknowledged their agency but questioned their subjectivity and the subject positions available to them. Girls and young women are constantly challenging boundaries, offering a counter discourse, and contributing to a shift in societal norms. This study adds further insight into how girls and women navigate these competing discourses about health, physical activity, and nutrition. To do so, girls and young women need to feel emotionally safe, and nature can potentially provide this important context.

## References

[cit0001] Azzarito, L. (2009). The panopticon of physical education: Pretty, active and ideally white. *Physical Education & Sport Pedagogy*, 14(1), 19–16. 10.1080/17408980701712106

[cit0002] Azzarito, L. (2010). Future Girls, transcendent femininities and new pedagogies: Toward girls’ hybrid bodies? *Sport, Education and Society*, 15(3), 261–275. 10.1080/13573322.2010.493307

[cit0003] Azzarito, L. (2012). Photography as a pedagogical tool for shedding light on ‘bodies-at-risk’ in physical culture. *Visual Studies*, 27(3), 295–309. 10.1080/1472586X.2012.717746

[cit0004] Azzarito, L., & Katzew, A. (2010). Performing identities in physical education. *Research Quarterly for Exercise and Sport*, 81(1), 25–37. 10.1080/02701367.2010.10599625 20387396

[cit0005] Azzarito, L., Solmon, M. A., & Harrison, L. (2006). “ … If i had a choice, i would … .” a feminist poststructuralist perspective on girls in physical education. *Research Quarterly for Exercise and Sport*, 77(2), 222–239. 10.1080/02701367.2006.10599356 16898278

[cit0006] Azzarito, L., & Sterling, J. (2010). ‘What it was in my eyes’: Picturing youths’ embodiment in ‘real’ spaces. *Qualitative Research in Sport and Exercise*, 2(2), 209–228. 10.1080/19398441.2010.488029

[cit0007] Barret, M. J. (2005). Making (some) sense of feminist poststructuralism in environmental education research and practice. *The Canadian Journal of Environmental Education*, 10(1), 79–93.

[cit0008] Boniface, M. (2007). The meaning of adventurous activities for ‘women in the outdoors’. *Journal of Adventure Education & Outdoor Learning*, 6(1), 9–24. 10.1080/14729670685200711

[cit0009] Bordo, S. (2003). *Unbearable weight: Feminism, western culture, and the body* (Tenth Anniversary ed.). University of California Press.

[cit0010] Boyce, W., King, M., & Roche, J. (2008). *Healthy settings for young people in Canada*. Public Health Agency of Canada.

[cit0011] Brazg, T., Bekemeier, B., Spigner, C., & Huebner, C. E. (2011). Our community in focus the use of photovoice for youth-driven substance abuse assessment and health promotion. *Health Promotion Practice*, 12(4), 502–511. 10.1177/1524839909358659 21051323

[cit0012] Brown, J. D., & Witherspoon, E. M. (2002). The mass media and American adolescents’ health. *Journal of Adolescent Health*, 31(6), 153–170. 10.1016/S1054-139X(02)00507-4 12470911

[cit0013] Bushnik, T. (2016). *Women in Canada: A gender-based statistical report (No. 89-503– X)*. Statistics Canada.

[cit0014] Butler, J. (1993). *Bodies that matter: On the discursive limits of “sex*. Routledge.

[cit0015] Canadian Minister of Finance. (2018). *Equality & growth: A strong middle class*. Her Majesty the Queen.

[cit0016] Carey, R. N., Donaghue, N., & Broderick, P. (2014). Body image concern among Australian adolescent girls: The role of body comparisons with models and peers. *Body Image*, 11(1), 81–84. 10.1016/j.bodyim.2013.09.006 24148894

[cit0017] Catalani, C., & Minkler, M. (2010). Photovoice: A review of the literature in health and public health. *Health Education & Behavior*, 37(3), 424–451. 10.1177/1090198109342084 19797541

[cit0018] Catherall, D. R. (2006). *Emotional safety: Viewing couples through the lens of affect*. Routledge Taylor & Francis Group. 10.4324/9780203961544

[cit0019] Cleland, J., 2005. Asking young people about sexual and reproductive behaviours [WWW document]. World Health Organ. (accessed 12 August 2017). http://www.who.int/reproductivehealth/topics/adolescence/questionnaire/en/

[cit0020] Cockburn, C., & Clarke, G. (2002). “Everybody’s looking at you!”: Girls negotiating the “femininity deficit” they incur in physical education. *Women’s Studies International Forum*, 25(6), 651–665. 10.1016/S0277-5395(02)00351-5

[cit0021] Dooley, J., 2016. Young, wild, and female: Gendered experiences at an outdoor adventure camp. Honors Theses AY 1516, University of Wyoming.

[cit0022] Doucet, A., & Mauthner, N. S. (2006). *Feminist methodoligies and epistemologies, in: Handbook of 21st century sociology*. Sage Publications.

[cit0023] Downing, L. (2008). *The Cambridge introduction to Michel Foucault*. Cambridge University Press.

[cit0024] Duncan, M. C. (1994). The politics of women’s body images and practices: Foucault, the panopticon, and shape magazine. *Journal of Sport and Social Issues*, 18(1), 48–65. 10.1177/019372394018001004

[cit0025] Eisenberg, M. E., & Neumark-Sztainer, D. (2010). Friends’ dieting and disordered eating behaviors among adolescents five years later: findings from project EAT. *Journal of Adolescent Health*, 47(1), 67–73. 10.1016/j.jadohealth.2009.12.030 20547294

[cit0026] Escobar-Chaves, S. L., & Anderson, C. A. (2008). Media and risky behaviors. *The Future of Children*, 18(1), 147–180. 10.1353/foc.0.0007 21338009

[cit0027] Evans, R. G. (2006). Fat zombies, pleistocene tastes, autophilia and the “obesity epidemic. *Healthcare Policy = Politiques De Sante*, 2(2), 18–26. doi: 10.12927/hcq..18653. 19305700 PMC2585437

[cit0028] Federal, provincial and territorial governments, 2018. A common vision for increasing physical activity and reducing sedentary living in Canada: Let’s get moving.

[cit0029] Felluga, D. F. (2015). *Critical theory: The key concepts* (1 ed.). Routledge.

[cit0030] Findholt, N. E., Michael, Y. L., & Davis, M. M. (2011). Photovoice engages rural youth in childhood obesity prevention. Public Health Nursing *Boston Mass*, 28(2), 186–192. 10.1111/j.1525-1446.2010.00895.x 21732973

[cit0031] Fitzgerald, A., Fitzgerald, N., & Aherne, C. (2012). Do peers matter? A review of peer and/or friends’ influence on physical activity among American adolescents. *Journal of Adolescence*, 35(4), 941–958. 10.1016/j.adolescence.2012.01.002 22285398

[cit0032] Flintoff, A., & Scraton, S. (2001). Stepping into active leisure? Young women’s perceptions of active lifestyles and their experiences of school physical education. *Sport, Education and Society*, 6(1), 5–21. 10.1080/713696043

[cit0033] Foucault, M. (1995). *Discipline & punish: The birth of the prison* (2nd ed.). Random House, Inc..

[cit0034] Freire, P. (2000). *Pedagogy of the oppressed* (30th anniversary ed.). Continuum.

[cit0035] Fung, C., McIsaac, J.-L. D., Kuhle, S., Kirk, S. F. L., & Veugelers, P. J. (2013). The impact of a population-level school food and nutrition policy on dietary intake and body weights of Canadian children. *Preventive Medicine*, 57(6), 934–940. 10.1016/j.ypmed.2013.07.016 23891787 PMC3842499

[cit0036] Gerbensky-Kerber, A. (2011). Grading the “good” body: A poststructural feminist analysis of body mass index initiatives. *Health Communication*, 26(4), 354–365. 10.1080/10410236.2010.551581 21416419

[cit0037] Guthold, R., Stevens, G. A., Riley, L. M., & Bull, F. C. (2018). Worldwide trends in insufficient physical activity from 2001 to 2016: A pooled analysis of 358 population-based surveys with 1·9 million participants. *The Lancet Global Health*, 6(10), e1077–e1086. 10.1016/S2214-109X(18)30357-7 30193830

[cit0038] Hancock, T. (2015). Population health promotion 2.0: An eco-social approach to public health in the anthropocene. *Canadian Journal of Public Health*, 106(4), 252–255. 10.17269/cjph.106.5161 PMC6972308 26285199

[cit0039] Rootman, I., Pederson, A., Frohlich, K., & Dupere, S. (2017). *Health Promotion in Canada*, Fourth Edition (4th ed.), pp. 408-433. Canadian Scholars’ Press.

[cit0040] *Health in 2015: From MDGs, millennium development goals to SDGs, sustainable development goals*. (2015). World Health Organization.

[cit0041] Hernandez, K., Engler-Stringer, R., Kirk, S., Wittman, H., & McNicholl, S. (2018). The case for a Canadian national school food program. *Canadian Food Studies/La Revue canadienne des études sur l’alimentation*, 5, 208–229. 10.15353/cfs-rcea.v5i3.260

[cit0042] Hesse-Biber, S. N. (2012). Feminist research: Exploring, interrogating, and transforming the interconnections of epistemology, methodology, and method. In *Handbook of Feminist Research*, 2-26. Sage Publications.

[cit0043] Kirk, S. F., & Ruetz, A., 2018. How to make a national school food program happen. The Conversation.

[cit0044] Krebs, N., Bagby, S., Bhutta, Z. A., Dewey, K., Fall, C., Gregory, F., Hay, W., Rhuman, L., Caldwell, C. W., & Thornburg, K. L. (2017). International summit on the nutrition of adolescent girls and young women: Consensus statement. *Annals of the New York Academy of Sciences*, 1400(1), 3–7. 10.1111/nyas.13417 28722768 PMC5601188

[cit0045] Kreuter, M. W., Rosa, C. D., Howze, E. H., & Baldwin, G. T. (2004). Understanding wicked problems: A key to advancing environmental health promotion. *Health Education & Behavior*, 31(4), 441–454. 10.1177/1090198104265597 15296628

[cit0046] Landman, M. (2006). Getting quality in qualitative research: A short introduction to feminist methodology and methods. *Proceedings of the Nutrition Society*, 65(4), 429–433. 10.1079/PNS2006518 17181910

[cit0047] Mackey, E. R., & Greca, A. M. L. (2007). Adolescents’ eating, exercise, and weight control behaviors: Does peer crowd affiliation play a role? *Journal of Pediatric Psychology*, 32(1), 13–23. 10.1093/jpepsy/jsl041 17093008

[cit0048] Mansfield, L., & Rich, E. (2013). Public health pedagogy, border crossings and physical activity at every size. *Critical Public Health*, 23(3), 356–370. 10.1080/09581596.2013.783685

[cit0049] Massa, C., 2015. Wild women: The positive transformation of women and girls through female-only adventure education. Undergrad. Honors Theses, University of Colorado.

[cit0050] Mâsse, L. C., Niet-Fitzgerald, J. E., De, Watts, A. W., Naylor, P.-J., & Saewyc, E. M. (2014). Associations between the school food environment, student consumption and body mass index of Canadian adolescents. *International Journal of Behavioral Nutrition and Physical Activity*, 11(1), 29. 10.1186/1479-5868-11-29 PMC3987130 24666770

[cit0051] McNiel, J. N., Harris, D. A., & Fondren, K. M. (2012). Women and the wild: Gender socialization in wilderness recreation advertising. *Gender Issues*, 29(1–4), 39–55. 10.1007/s12147-012-9111-1

[cit0052] National Eating Disorder Information Centre. (2015). *Research suggests that eating disorders are more common than previously thought* (Vol. Press Release). NEDIC.

[cit0053] Numer, M. S., & Gahagan, J. (2009). The sexual health of gay men in the post-AIDS era: Feminist, post-structuralist and queer theory perspectives. *International Journal of Men’s Health*, 8(2), 155–168. 10.3149/jmh.0802.155

[cit0054] Overholt, J. R., & Ewert, A. (2015). Gender matters: exploring the process of developing resilience through outdoor adventure. *The Journal of Experimental Education*, 38(1), 41–55. 10.1177/1053825913513720

[cit0055] ParticipACTION. (2018). *The brain + body equation: Canadian kids need active bodies to build their best brains. The 2018 participation report card on physical activity for children and youth*.

[cit0056] Pate, R. R., Trilk, J. L., Byun, W., & Wang, J. (2011). Policies to increase physical activity in children and youth. *Journal of Exercise Science and Fitness*, 9(1), 1–14. 10.1016/S1728-869X(11)60001-4

[cit0057] Patte, K. A., Laxer, R. E., Qian, W., & Leatherdale, S. T. (2016). An analysis of weight perception and physical activity and dietary behaviours among youth in the COMPASS study. *SSM - Population Health*, 2, 841–849. 10.1016/j.ssmph.2016.10.016 29349193 PMC5757788

[cit0058] Rancourt, D., Choukas-Bradley, S., Cohen, G. L., & Prinstein, M. J. (2014). An experimental examination of peers’ influence on adolescent girls’ intent to engage in maladaptive weight-related behaviors. *International Journal of Eating Disorders*, 47(5), 437–447. 10.1002/eat.22258 PMC4624103 24482093

[cit0059] REI, 2017. 2017 national study on women and the outdoors [WWW document]. https://www.slideshare.net/REI_/2017-national-study-on-women-and-the-outdoors

[cit0060] Rich, E., & Evans, J. (2005). Making sense of eating disorders in schools. *Discourse Studies in the Cultural Politics of Education*, 26(2), 247–262. 10.1080/01596300500143211

[cit0061] Rich, E., & Evans, J. (2013). Changing times, future bodies? The significance of health in young women’s imagined futures. *Pedagogy, Culture & Society*, 21(1), 5–22. 10.1080/14681366.2012.748680

[cit0062] Riediger, N. D., Shooshtari, S., & Moghadasian, M. H. (2007). The influence of sociodemographic factors on patterns of fruit and vegetable consumption in Canadian adolescents. *Journal of the American Dietetic Association*, 107(9), 1511–1518. 10.1016/j.jada.2007.06.015 17761228

[cit0063] Rigg, K. K., Cook, H. H., & Murphy, J. W. (2014). Expanding the scope and relevance of health interventions: Moving beyond clinical trials and behavior change models. *International Journal of Qualitative Studies on Health and Well-being*, 9(1), 24743. 10.3402/qhw.v9.24743 25053530 PMC4107301

[cit0064] Roberts, K. C., Yao, X., Carson, V., Chaput, J.-P., Janssen, I., & Tremblay, M. S. (2017). Meeting the Canadian 24-hour movement guidelines for children and youth. *Health Reports*, 28(10), 3–7. PMID: 29044440. 29044440

[cit0065] Royce, S. W., Parra-Medina, D., & Messias, D. H. (2006). Using photovoice to examine and initiate youth empowerment in community-based programs: A picture of process and lessons learned. *Californian Journal of Health Promotion*, 4(3), 80–91. 10.32398/cjhp.v4i3.1960

[cit0066] Sackett, C. R., Newhart, S., Jenkins, A. M., & Cory, L. (2018). Girls’ perspectives of barriers to outdoor physical activity through photovoice: A call for counselor advocacy. *Journal of Creativity in Mental Health*, 13(1), 2–18. 10.1080/15401383.2017.1343166

[cit0067] Sailors, P. R., Teetzel, S., & Weaving, C. (2012). No Net Gain: A critique of media representations of women’s Olympic beach volleyball. *Feminist Media Studies*, 12(3), 468–472. 10.1080/14680777.2012.698093

[cit0068] Shea, J., Poudrier, J., Chad, K., & Atcheynum, J. (2011). Understanding the healthy body from the perspective of first nations girls in the Battlefords Tribal Council Region: A photovoice project. *Native Studies Review*, 20(1), 27–57.

[cit0069] Signal, L. N., Walton, M. D., Mhurchu, C. N., Maddison, R., Bowers, S. G., Carter, K. N., Gorton, D., Heta, C., Lanumata, T. S., McKerchar, C. W., O’Dea, D., & Pearce, J. (2013). Tackling ‘wicked’ health promotion problems: A New Zealand case study. *Health Promotion International*, 28(1), 84–94. 10.1093/heapro/das006 22419621

[cit0070] Slater, A., & Tiggemann, M. (2011). Gender differences in adolescent sport participation, teasing, self-objectification and body image concerns. *Journal of Adolescence*, 34(3), 455–463. 10.1016/j.adolescence.2010.06.007 20643477

[cit0071] Spencer, R. A., Rehman, L., & Kirk, S. F. (2015). Understanding gender norms, nutrition, and physical activity in adolescent girls: A scoping review. *International Journal of Behavioral Nutrition and Physical Activity*, 12(1), 6. 10.1186/s12966-015-0166-8 PMC4310022 25616739

[cit0072] Standiford, A. (2013). The secret struggle of the active girl: A qualitative synthesis of interpersonal factors that influence physical activity in adolescent girls. *Health Care for Women International*, 34(10), 860–877. 10.1080/07399332.2013.794464 23790150

[cit0073] Strack, R. W., Magill, C., & McDonagh, K. (2004). Engaging youth through photovoice. *Health Promotion Practice*, 5(1), 49–58. 10.1177/1524839903258015 14965435

[cit0074] Strasburger, V. C., Jordan, A. B., & Donnerstein, E. (2010). Health effects of media on children and adolescents. *Pediatrics*, 125(4), 756–767. 10.1542/peds.2009-2563 20194281

[cit0075] Temmerman, M., Khosla, R., Laski, L., Mathews, Z., & Say, L. (2015). Women’s health priorities and interventions. *BMJ*, 35 (Suppl1), h4147. 10.1136/bmj.h4147 26371215

[cit0076] Vaughn, L. M., Rojas-Guyler, L., & Howell, B. (2008). “Picturing” health: A photovoice pilot of Latina girls’ perceptions of health. *Family & Community Health*, 31(4), 305–316. 10.1097/01.FCH.0000336093.39066.e9 18794637

[cit0077] Wang, C. Photovoice: A participatory action research strategy applied to women’s health. (1999). *Journal of Women’s Health*, 8(2), 185–192. 8p. 10.1089/jwh.1999.8.185 10100132

[cit0078] Wang, C. (2006). Youth participation in photovoice as a strategy for community change. *Journal of Community Practice*, 14(1–2), 147–161. 10.1300/J125v14n01_09

[cit0079] Wang, C., & Burris, M. A. (1997). Photovoice: Concept, methodology, and use for participatory needs assessment. *Health Education and Behaviour*, 24(3), 369–387. 10.1177/109019819702400309 9158980

[cit0080] Wang, C., & Pies, C. A. (2004). Family, maternal, and child health through photovoice. *Maternal and Child Health Journal*, 8(2), 95–102. 10.1023/B:MACI.0000025732.32293.4f 15198177

[cit0081] Wang, C., & Redwood-Jones, Y. A. (2001). Photovoice ethics: Perspectives from flint photovoice. *Health Education & Behavior*, 28(5), 560–572. 10.1177/109019810102800504 11575686

[cit0082] Wang, C., Yi, W. K., Tao, Z. W., & Carovano, K. (1998). Photovoice as a participatory health promotion strategy. *Health Promotion International*, 13(1), 75–86. 10.1093/heapro/13.1.75

[cit0083] Ward, P. R., Meyer, S. B., Verity, F., Gill, T. K., & Luong, T. C. (2011). Complex problems require complex solutions: The utility of social quality theory for addressing the social determinants of health. *BMC Public Health*, 11(1), 630. 10.1186/1471-2458-11-630 21819576 PMC3167771

[cit0084] Weedon, C. (1987). *Feminist Practice and Poststructuralist Theory*. Basil-Blackwell.

[cit0085] Weitz, R., 2002. A history of women’s bodies [WWW document]. http://www.fwhc.org/roseweitz1.htm (accessed July 20 2016). Feminist Women's Health Centre.

[cit0086] Wesely, J. K., & Gaarder, E. (2004). The gendered “nature” of the urban outdoors: Women negotiating fear of violence. *Gender & Society*, 18(5), 645–663. 10.1177/0891243204268127

[cit0087] Whittington, A. (2006). Challenging girls’ constructions of femininity in the outdoors. *The Journal of Experimental Education*, 28(3), 205–221. 10.1177/105382590602800304

[cit0088] Williams, A. J., Henley, W. E., Williams, C. A., Hurst, A. J., Logan, S., & Wyatt, K. M. (2013). Systematic review and meta-analysis of the association between childhood overweight and obesity and primary school diet and physical activity policies. *International Journal of Behavioral Nutrition and Physical Activity*, 10(1), 101. 10.1186/1479-5868-10-101 PMC3844408 23965018

[cit0089] Witmer, L., Bocarro, J. N., & Henderson, K. (2011). Adolescent girls’ perception of health within a leisure context. *Journal of Leisure Research*, 43(3), 334–354. 10.1080/00222216.2011.11950240

[cit0090] World Health Organization, 2014. Global strategy on diet, physical activity and health [WWW document]. World Health Organ. http://www.who.int/dietphysicalactivity/pa/en/index.html

[cit0091] Wright, J., O’Flynn, G., & Macdonald, D. (2006). Being fit and looking healthy: young women’s and men’s constructions of health and fitness. *Sex Roles*, 54(9–10), 707–716. 10.1007/s11199-006-9036-9

